# *Mycoplasma pneumoniae*-associated angioedema

**DOI:** 10.1016/j.jdcr.2021.01.004

**Published:** 2021-01-17

**Authors:** Patrick M. Meyer Sauteur, Martin Theiler, Bettina Bogatu

**Affiliations:** a Division of Infectious Diseases and Hospital Epidemiology, University Children's Hospital Zurich, Zurich, Switzerland; b Pediatric Skin Center–Department of Dermatology, University Children's Hospital Zurich, Zurich, Switzerland; c Division of Allergology, University Children's Hospital Zurich, Zurich, Switzerland

**Keywords:** allergy, anaphylaxis, drug-induced, *Mycoplasma pneumoniae*-induced rash and mucositis (MIRM), urticarial, AE, angioedema, CAP, community-acquired pneumonia

## Introduction

Angioedema (AE) is a self-limiting and benign condition, but may present as a medical emergency due to upper airway obstruction.^1^ Different pathological processes involving proinflammatory mediators cause several distinct subtypes of AE.^2^ To our knowledge, we here report the first case of *Mycoplasma pneumoniae*-associated AE in a child. Recognition of this clinical entity prevents extensive diagnostic testing and avoids restriction of possibly causative drugs.

## Case report

A previously healthy 5-year-old boy presented with a cough and fever since 1 week. On clinical examination, he was tachypneic and had crackles on the right hemithorax. Laboratory investigations revealed hemolytic anemia (10.9 g/dL; range, 11.5-14.0), normal white blood cell count (6.8 × 10^9^/L) without eosinophilia, and an elevated C-reactive protein level (22.0 mg/L; normal level <10.0). A chest radiograph showed bilateral interstitial infiltrates with small pleural effusions on both sides. *M pneumoniae* was detected by polymerase chain reaction of pharyngeal swab samples and strongly positive *M pneumoniae*-specific IgM (>150 U/mL; cutoff, 17 U/mL) and IgG (89 U/mL; cutoff, 15 U/mL) (Virion/Serion) serum antibody levels. A diagnosis of *M pneumoniae* community-acquired pneumonia (CAP) was made. He was started on oral clarithromycin with 15 mg/kg/day in 2 doses.

**Fig 1:**
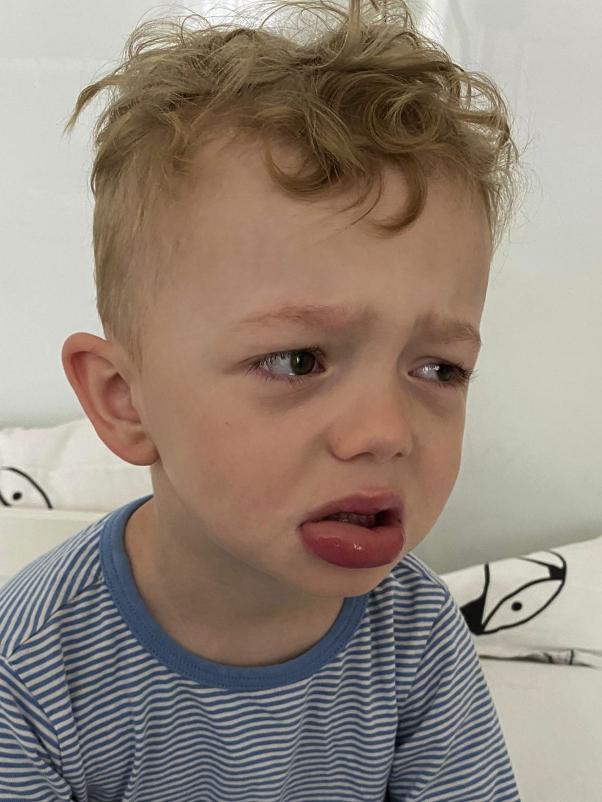
*Mycoplasma pneumoniae*-associated angioedema of the lower lip.

Following the administration of the third dose in the evening, he awoke in the morning with massive angioedematous swelling of the lower lip (Fig 1). He did not complain about dyspnea or pruritus, and did not show any accompanying tongue swelling. Erosions or involvement of other mucosal surfaces were absent, as were skin lesions such as wheals or target-like papules and plaques. No other drugs apart from clarithromycin were administered. He received a single dose of oral levocetirizine and betamethasone for the differential diagnosis of histamine-mediated AE. The swelling resolved slowly within a day. A diagnosis of AE was made.^2^^,^^3^ *M pneumoniae* infection was further confirmed with the detection of specific IgM antibody-secreting cells by enzyme-linked immunospot (ELISpot) assay.^4^^,^^5^ Other infectious triggers, ie, herpes simplex virus, hepatitis B virus, Epstein-Barr virus, cytomegalovirus, and parvovirus B19 were excluded by serology.

There was no recurrence following restart of clarithromycin for a total of 7 days or after drug challenge with clarithromycin (to exclude drug-induced AE) at a 6-week follow-up. At this time point, he had fully recovered from *M pneumoniae* CAP, and laboratory investigations revealed normal C4 level and C1-inhibitor level and function (to exclude hereditary AE) and normal serum tryptase levels (to exclude systemic mastocytosis). *M pneumoniae*-specific IgM (>150 U/mL) and IgG (127 U/mL) antibodies remained at very high levels, which additionally supports an infection-triggered process elicited by *M pneumoniae* as cause of AE.

## Discussion

AE is localized and non-pruritic swelling of submucosal or subcutaneous tissue due to vasodilatation and increased vascular permeability.^2^ It involves predominantly the tongue, lip, face, mouth, throat, and extremities. Laryngeal involvement may cause upper airway obstruction and be fatal if not addressed promptly. The diagnosis of AE is made clinically based on a suggestive history and physical findings.^3^ AE can be classified into mainly idiopathic (no underlying cause identified), IgE-mediated (allergic), bradykinin-mediated (hereditary AE and ACE inhibitor-induced), and infection-triggered AE.^1^ Most frequently reported infectious triggers are viral pathogens, such as herpes simplex virus, coxsackie A and B virus, hepatitis B virus, and Epstein-Barr virus.^1^^,^^2^

*M pneumoniae* can cause a wide range of mucocutaneous manifestations,^6^ mainly maculopapular skin eruptions, urticaria, and *M pneumoniae*-induced rash and mucositis.^7^ *M pneumoniae*-induced rash and mucositis may present with lip swelling at disease onset, but is defined as a mucosal-predominant rash with wide-spread erosions on at least 2 mucosal sites, frequently accompanied by a few scattered targetoid lesions^8^—features that were clearly absent in our patient. Although we observed urticaria in a recent series among 4.5% of children with *M pneumoniae* CAP,^7^ AE in association with *M pneumoniae*, to our knowledge, has only been reported in a single adult case.^9^ Pathophysiological considerations for *M pneumoniae*-associated AE include immune complex formation by cold agglutinins that acts as anaphylatoxins.^2^^,^^9^ The hemolytic anemia in our patient with *M pneumoniae* CAP during the onset of AE may also suggest a potential cold agglutinin-related disease.

In conclusion, the confirmed diagnosis of *M pneumoniae* infection in this case together with the exclusion of other known causes of AE (including other infections) supports the role of *M pneumoniae* infection as a specific trigger of AE. *M pneumoniae* is known to cause a variety of dermatological manifestations. This report adds *M pneumoniae* to reactive infectious causes leading to parainfectious (secondary) AE.

## Conflicts of interest

None disclosed.
